# Loss of the mitochondrial calcium‐sensor, MICU3, contributes to Alzheimer’s disease pathogenesis

**DOI:** 10.1002/alz.095245

**Published:** 2025-01-09

**Authors:** Tyler Lewis Stevens, Henry M Cohen, Oniel Salik, Pooja Jadiya, Luke Daniel Sauers, Anya Wilkinson, John W Elrod

**Affiliations:** ^1^ Temple University, Philadelphia, PA USA; ^2^ Wake Forest University School of Medicine, Winston‐Salem, NC USA

## Abstract

**Background:**

While the formation of β‐amyloid plaques and neurofibrillary “tau” tangles are considered hallmarks of AD pathology, therapeutic targeting of these pathways has been unsuccessful, highlighting the necessity to define the underlying molecular mechanisms driving AD progression. Previous studies from our lab demonstrated that mitochondrial calcium (_m_Ca^2+^) overload through neuronal ablation of the mitochondrial Na^+^/Ca^2+^ exchanger (NCLX) is sufficient to trigger ‘AD‐like’ pathology, including mitochondrial dysfunction, amyloid deposition and tau pathology, and cognitive decline. In addition, we found significant proteomic remodeling of components of the mitochondrial calcium uniporter channel (mtCU), the primary mediator of _m_Ca^2+^ uptake, in frontal cortex samples isolated post‐mortem from patients diagnosed with non‐familial/sporadic AD. Specifically, our results indicate reduced neuronal expression of MICU3, an EF‐hand containing protein reported to regulate the mtCU, is AD patients and animal models of AD. Here we tested the hypothesis that loss of neuronal MICU3 contributes to AD pathogenesis through perturbed _m_Ca^2+^ signaling.

**Method:**

To examine the role of MICU3 in AD progression, we developed a neuronal‐specific knockout model of MICU3 (nKO‐MICU3) and crossbred this to multiple AD mouse models, including the APP^NL‐G‐F^ genetic knock‐in model and the transgenic 5xFAD model. Behavioral experiments, including y‐maze alternations, novel object recognition and contextual recall fear conditioning were each performed at three separate timepoints. MICU3 was also ablated from N2a cells to characterize calcium parameters.

**Result:**

The loss of neuronal MICU3 alone is sufficient to impair learning and memory, deficits that are comparable to robust genetic AD models. Interestingly, nKO‐MICU3 mice displayed impaired contextual fear memory at an earlier age than AD models, suggesting the loss of MICU3 is sufficient to produce an AD‐like phenotype. Indeed, loss of MICU3 increased neuronal cell death during calcium stress, and also impaired _m_Ca^2+^ uptake rates.

**Conclusion:**

Loss of MICU3 is sufficient to induce an AD‐like phenotype. Our findings highlight the necessity to further explore how mitochondrial dysfunction may trigger the formation and/or progression of AD. Future goals define the molecular pathophysiology associated with loss of MICU3, as well as determining if rescuing neuronal MICU3 expression is sufficient to mitigate or reverse neuropathology and cognitive decline in various mutant AD models.

